# Comparative Transcriptome Analysis of Resistant and Susceptible Tomato Lines in Response to Infection by *Xanthomonas perforans* Race T3

**DOI:** 10.3389/fpls.2015.01173

**Published:** 2015-12-24

**Authors:** Heshan Du, Yuqing Wang, Jingjing Yang, Wencai Yang

**Affiliations:** Beijing Key Laboratory of Growth and Developmental Regulation for Protected Vegetable Crops, Department of Vegetable Science, China Agricultural UniversityBeijing, China

**Keywords:** *Solanum lycopersicum*, *Xanthomonas perforans*, compatible and incompatible interactions, transcriptome analysis, RNA sequencing

## Abstract

Bacterial spot, incited by several *Xanthomonas* sp., is a serious disease in tomato (*Solanum lycopersicum* L.). Although genetics of resistance has been widely investigated, the interactions between the pathogen and tomato plants remain unclear. In this study, tanscriptomes of *X. perforans* race T3 infected tomato lines were compared to those of controls. An average of 7 million reads were generated with approximately 21,526 genes mapped in each sample post-inoculation at 6 h (6 HPI) and 6 days (6 DPI) using RNA-sequencing technology. Overall, the numbers of differentially expressed genes (DEGs) were higher in the resistant tomato line PI 114490 than in the susceptible line OH 88119, and the numbers of DEGs were higher at 6 DPI than at 6 HPI. Fewer genes (78 in PI 114490 and 15 in OH 88119) were up-regulated and most DEGs were down-regulated, suggesting that the inducible defense response might not be fully activated at 6 HPI. Accumulation expression levels of 326 co-up regulated genes in both tomato lines at 6 DPI might be involved in basal defense, while the specific and strongly induced genes at 6 DPI might be correlated with the resistance in PI 114490. Most DEGs were involved in plant hormone signal transduction, plant-pathogen interaction and phenylalanine metabolism, and the genes significantly up-regulated in PI 114490 at 6 DPI were associated with defense response pathways. DEGs containing NBS-LRR domain or defense-related WRKY transcription factors were also identified. The results will provide a valuable resource for understanding the interactions between *X. perforans* and tomato plants.

## Introduction

Bacterial spot caused by at least four distinct species of *Xanthomonas* (*X. euvesicatoria, X. vesicatoria, X. perforans*, and *X. gardneri*), severely affects marketability of both fresh-market and processing tomato (Jones et al., 2000; Stall et al., 2009). Based on their virulence on a group of tomato genotypes, the causative agents of bacterial spot can be classified into five physiological races T1–T5 (Jones et al., 2005). It is difficult to manage the disease once the outbreak occurs (Yang et al., 2007; Stall et al., 2009). Identification and characterization of the host resistance as well as regulatory genes can provide insights into understanding the mechanisms of host resistance and the use of host resistance to develop new cultivars with durable resistance.

The resistance to *X. perforans* race T3 in tomato can be classified into two types, hypersensitive response (HR) and field resistance. HR is usually conditioned by single dominant genes, while field resistance is generally controlled by multiple genes (Yang, 2013). To date, four major dominant genes, *Rx4* in *Solanum pimpinellifolium* accession PI 128216 (Robbins et al., 2009; Pei et al., 2012), *Xv3* in an unimproved breeding line Hawaii 7981 (Wang et al., 2011), *Rx*_*LA*1589_ in *S. pimpinellifolium* accession LA 1589 (Sun et al., 2011a), and *RXopJ4* in *S. pennellii* accession LA 716 (Sharlach et al., 2013), conferring HR to *X. perforans* race T3 have been identified and mapped. A recent study suggests that *Rx4, Xv3*, and *Rx*_*LA*1589_ are possibly the same gene (Zhao et al., 2015). Although these genes also confer field resistance to race T3, they need modifiers and may be affected by genetic background (Scott et al., 2001; Robbins et al., 2009; Wang et al., 2011; Sharlach et al., 2013). Therefore, the tomato lines with HR usually show partial resistance to race T3 in the field. On the contrary, the *S. lycopersicum* var. *cerasiforme* accession PI 114490 shows no HR but high resistance to race T3 conditioned by at least five quantitative trait loci (QTL) in the field (Sun et al., 2011b, 2014; Scott et al., 2015). In addition to identification and genetic mapping of genes or QTLs, efforts have also been making on discovery of genes participating in a complex molecular network of regulation during the time-course of HR to race T3 in the unimproved tomato line Hawaii 7981 using microarray analysis approach (Gibly et al., 2004; Balaji et al., 2007), and identification of genes differentially expressed in the resistant line PI 114490 and a susceptible line OH 88119 during the time-course of the race T3 infection using cDNA-AFLP techniques (Du et al., 2014). Comparison of genes differentially expressed in PI 114490 and OH 88119 provides us some information to understand the mechanism of resistance to bacterial spot race T3 in tomato during the process of symptom development at the 3, 4, and 5 days post spray-inoculation stages (Du et al., 2014). However, both microarray and cDNA-AFLP can only identify part of the genes involved in resistance or defense processes due to their low throughput limitation. To fully unravel the mechanisms of field resistance to race T3 of bacterial spot, it is necessary to identify more genes differentially expressed during different infection times in tomato.

With the availability of a high-quality tomato genome sequence and second-generation sequencing, RNA-seq technology has rapidly become a popular tool for genome-wide expression profiling, providing the potential to better understanding the comprehensive host-pathogen interactions. Many studies have, therefore, tried to elucidate a nearly complete picture of inducible defense response pathways using RNA-seq analysis (Kim et al., 2011; Li et al., 2012; Gao et al., 2013; Tan et al., 2015). Through comparing the different RNA-seq data about hosts and pathogens interactions, similar or specific sets of genes activated in different plant pathosystems have been discovered. Meanwhile, using the expression pattern regulated by pathogen infection, it is also possible to identify the known defense gene family member involved in the tomato immune response. For example, some plant R genes, such as pepper *Bs3* and rice *Xa27* belonging to NBS-LRR gene family, are transcriptionally activated by corresponding transcription activator-like effector (TALE) protein (Gu et al., 2005; Röemer et al., 2007). Taking advantage of the TALE-induced expression pattern, a *Xanthomonas* TAL-effector activated resistance gene *Bs4C* has been identified using RNA-seq (Strauss et al., 2012). Therefore, RNA-seq technology can be used as a tool to isolate pathogen induced R gene, which could avoid laborious positional cloning and investigate the expression pattern of different defense signaling genes family members as well as identify novel defense related genes.

In the current study, it is first time using RNA-seq to investigate transcript dynamics of the field resistant line PI 114490 and the susceptible line OH 88119 in response to *X. perforans* race T3 at 6 h and 6 days post-infection. Large-scale expression profiling identified both common and specific differentially expressed genes in two tomato lines at different time points after inoculation. In addition, 8 induced genes were validated by quantitative real-time PCR (qRT-PCR) experiments. Our results may provide a subset of potential candidate defense-related genes in tomato-*X. perforans* interaction, which will help in better understanding the molecular basis of field resistance to bacterial spot in tomato.

## Materials and methods

### Plant materials and *X. perforans* race T3 inoculation

Two tomato lines, a cherry tomato line PI 114490 with high level of field resistance and an elite processing breeding line OH 88119 with susceptibility to *X. perforans* race T3 (Sun et al., 2011b), were used in this study. For inoculation with *X. perforans* race T3, tomato seedlings were grown in a growth room at 28°C/25°C (light/dark), with a 14-h photoperiod. The race T3 strain *Xv829* was cultured on yeast, dextrose, calcium carbonate (YDC) agar medium (Lelliot and Stead, 1987) at 28°C for 48–72 h prior to inoculation. Bacterial cultures were diluted to a concentration of approximately 3 × 10^8^ colony forming units per ml using sterile solution containing 10 mM MgSO_4_·7H_2_O and 0.025% (v/v) Silwet L77. Six-week-old plants were spray-inoculated with the bacterial suspension, and plants sprayed with the sterile solution containing 10 mM MgSO_4_·7H_2_O and 0.025% (v/v) Silwet L77 were used as controls (mock-treatment). The plants were misted with water twice a day (9:00 a.m. and 5:00 p.m.) from 1 day before inoculation to 6 days after inoculation to increase humidity and prolong leaf wetness for disease development.

### RNA isolation, RNA-seq library preparation, and sequencing

Total RNA was isolated from leaf samples collected at started point (mock-treatment), 6 h-post-inoculation (HPI) and 6 day-post-inoculation (DPI) using the TRIzol reagent (Invitrogen, USA) following to the manufacturer's protocol. The concentration of total RNA was determined by NanoDrop 2000 Spectrophotometer (Thermo Fisher Scientific, USA), and the RNA integrity value (RIN) was checked using RNA 6000 Pico LabChip of Agilent 2100 Bioanalyzer (Agilent, USA).

Three libraries (mock-treatment, race T3 treatment at 6 HPI and 6 DPI) for each tomato line were constructed for RNA-seq analysis. Two hundred micro-grams total RNA was incubated with 10 units DNase I (Ambion, USA) at 37°C for 1 h, and then nuclease-free water was added to bring the sample volume to 250 μl. The MicroPoly(A)Purist™ Kit (Ambion) was used to enrich mRNA according to the manufacture's protocol. The enriched mRNA was dissolved in 100 μl RNA storage solution and the final concentration was determined by NanoDrop 2000 Spectrophotometer. Double-stranded cDNA was synthesized from the mRNA according to the method described previously (Ng et al., 2005) with some modifications. First-strand cDNA was synthesized from 10 μg mRNA with Gsul-oligo dT primers using 1000 units of superscript II reverse transcriptase (Invitrogen). After incubation at 42°C for 1 h, the 5′-CAP structure of mRNA was oxidized by NalO_4_ (Sigma, USA) and ligated to biotin hydrazide, which was used to select complete mRNA/cDNA by binding Dynal M280 beads (Invitrogen). The second-strand cDNA synthesis was subsequently performed using Ex Taq polymerase (TaKaRa, Japan). The polyA and 5′ adaptor were removed by GsuI digestion. The double-strand cDNA was ultrasonically fractioned with a cDNA size fractionation column at a range of 300–500 bp, and then purified using Agencourt Ampure beads (Agencourt, USA). Sequencing libraries were generated using TruSeqTM DNA Sample Prep Kit—Set A (Illumina, USA). Libraries were clustered using TruSeq PE Cluster Kit (Illumina) according to manufacturer's instructions. After cluster generation, the libraries were sequenced with an Illumina HiSeq™ 2000 platform by Hanyu Bio-Tech Co., Ltd (Shanghai, China).

### Analysis of illumina reads and identification of differentially expressed genes

Raw reads were firstly cleaned by removing adaptor sequences and unreliable low quality sequences (reads with unknown sequences “N”). The filtered clean reads were then mapped to *S. lycopersicum* reference genome sequence (SL2.40 version) and related annotation was obtained from the International Tomato Annotation Group (ITAG) Release 2.3 Predicted CDS (https://www.sgn.cornell.edu/) using Bowtie2 with default settings (Langmead et al., 2009). Clean reads mapped to reference genome sequence with multiple genes were filtered. Reads per kilo bases per million uniquely mapped reads (RPKM) values per sample for all genes were calculated based on both the total number of reads that mapped to the tomato reference CDS database and corresponding genes length (Mortazavi et al., 2008). Differentially expressed genes (DEG) were identified by DEG-seq package using the MA-plot-based method with Random Sampling model (MARS) (Wang et al., 2010). The *q*-value was adjusted using the method described in Benjamini and Hochberg (1995). “False Discovery Rate (FDR) ≤ 0.001 and the absolute value of Log2fold-change ≥ 2” were used as the threshold to determine the significance of gene expression difference.

### Gene ontology and KEGG pathway analysis

Gene ontology analysis for DEGs was performed using GoPipe (Chen et al., 2005) through BLASTP against Swiss-Prot and TrEMBL database. The metabolic pathway was constructed based on Kyoto Encyclopedia of Genes and Genomes (KEGG) database using BBH (bi-directional best hit) method (Kanehisa et al., 2010). The KO number was obtained for each protein and used for constructing metabolic pathways.

### RT-PCR and qRT-PCR validation

To verify the results of RNA-seq analysis, 8 differentially expressed genes (Receptor like kinase, Polyphenol oxidase, Harpin-induced protein-like, LRR receptor-like serine/threonine-protein kinase, 1-aminocyclopropane-1-carboxylate oxidase, Calmodulin 2, Universal stress protein family protein, and WRKY transcription factor 16) were selected for semi-quantitative RT-PCR and qRT-PCR analysis. First strand cDNA was synthesized by M-MLV reverse transcriptase (TaKaRa) with the same batch of RNA samples used in RNA-seq analysis. The specificity of primers for each gene (Table S1) was confirmed by analyzing PCR products on agarose gel and melting curve during real-time PCR. RT-PCR and qRT-PCR was conducted according to the previous protocols (Pei et al., 2012; Du et al., 2014).

## Results

### Analysis of RNA-seq libraries and reads alignment

Approximately 43 million Illumina raw reads were generated from six libraries with an average of 7 million 100 bp raw reads in each individual library. The raw reads were deposited in the National Center for Biotechnology Information Sequence Read Archive under the accession number SRP065597. In the six libraries, 97.9–99.3% of raw reads were clean. Of the clean reads, 46.5–77.3% could be mapped uniquely to one location within the tomato reference genome, 8.4–40.3% could be aligned to highly conserved domain sequences shared by different annotated genes, and 9.6–21.3% failed to map to the tomato genome sequence (Table 1). The uniquely mapped clean reads matched 21,168–21,993 with an average of 21,526 genes (approximately 62%) to the tomato annotated genes for the six libraries (Table 1). Approximately 20,000 genes were mapped at both mock- and T3-treatment samples (Image S1). The number of specific matched genes was higher in the PT6d library (890) than in other five libraries (Image S1).

**Table 1:** **Summary of RNA-seq data**.

**Sample ID^a^**	**No. of raw reads**	**No. of clean reads**				**No. of matched genes**
		**Total**	**Mapped to unique genes**	**Mapped to multiple genes**	**Unmapped**	
PM	10,937,043	10,783,924	5,402,226	4,347,202	1,034,496	21,776
PT6h	6,707,580	6,603,613	4,852,118	556,598	1,194,897	21,345
PT6d	7,148,378	7,096,195	5,421,494	548,184	1,126,517	21,993
OM	6,154,320	6,094,623	4,710,354	476,999	907,270	21,708
OT6h	5,381,872	5,316,213	3,927,090	399,894	989,229	21,168
OT6d	6,921,528	6,773,407	3,148,353	2,181,678	1,443,376	21,168
Average	7,208,454	7,111,329	4,576,939	1,418,426	1,115,964	21,526

a *PM and OM: PI 114490 and OH 88119 respectively mock-treatment with the sterile solution containing 10 mM MgSO_4_. 7H_2_O and 0.025%(v/v) Silwet L77. PT and OT: PI 114490 and OH 88119 respectively inoculated with bacterial spot race T3*.

The genes RPKM values were calculated only for those uniquely mapped reads. The number of genes with RPKM values of 0 varied from 12,734 to 13,559, with an average of 13,200 in the six libraries (Table S2). Of which 10,117 were common in all libraries, indicating a very low level or no expression of those genes in the six samples. The numbers of genes with RPKM value of 0 were slightly higher in the three libraries of the susceptible line OH 88119 than the corresponding samples in the resistant line PI 114490. Based on the expression analysis using RT-PCR and qRT-PCR, genes with RPKM value less than 2.28 needed more amount of cDNA and genes with RPLM value greater than 10.7 needed less amount of cDNA for obtaining clear bands (data not shown). Therefore, genes with RPKM values of 0–3 were considered as at low level of expression, while genes with RPKM values beyond 10 were considered to be expressed at high level. The number of highly expressed genes was less in PT6h library compared to OT6h, but more in PT6d than OT6d (Table S2).

### Differentially expressed genes in response to *X. perforans* race T3

**Figure 1:**
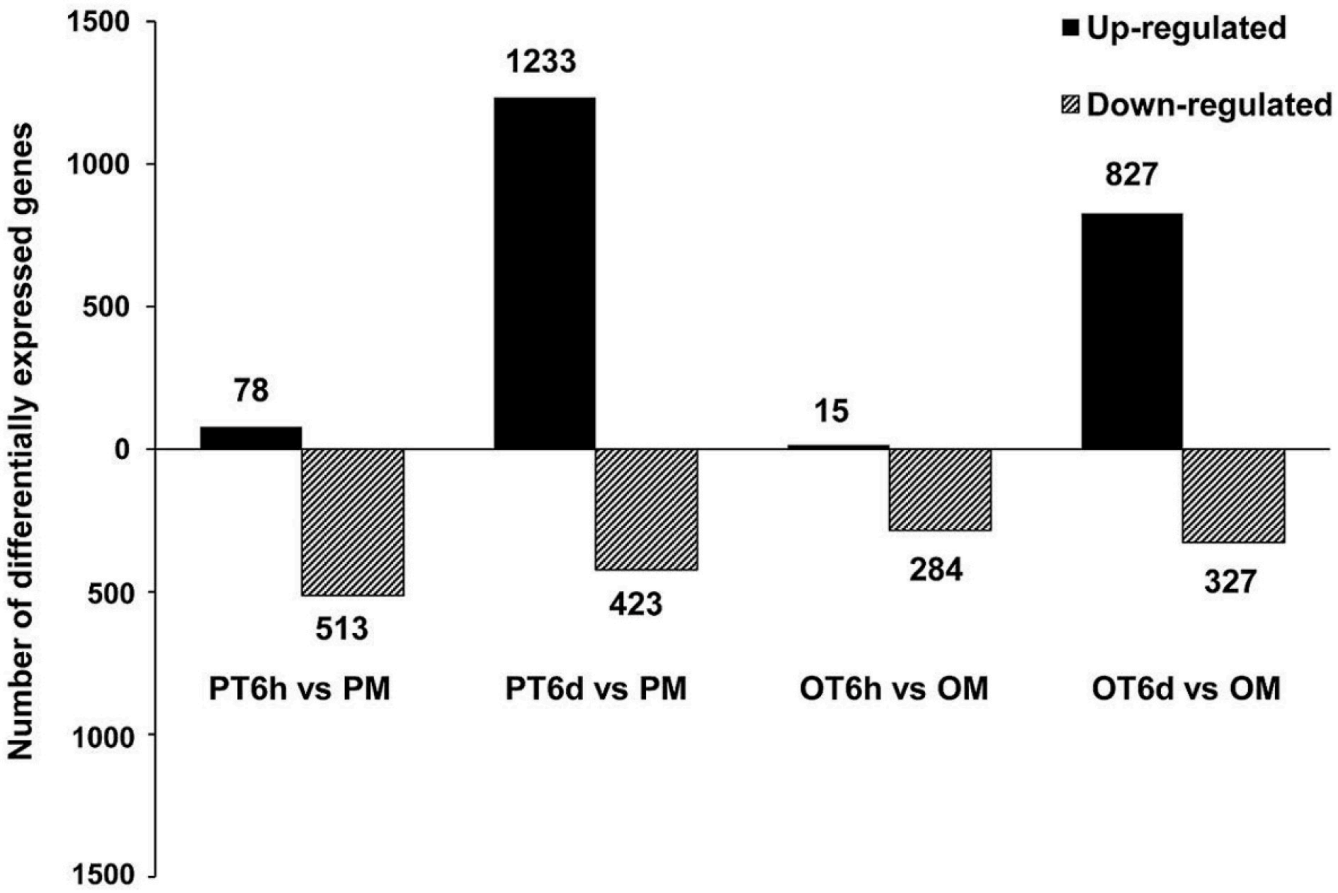
**Numbers of differentially expressed genes (DEGs) identified in tomato lines PI 114490 and OH 88119 at 6 h and 6 days post-inoculation of *Xanthomonas perforans* race T3**. The DEGs were identified using FDR ≤ 0.001 and the absolute value of Log_2_ fold-change ≥ 2 as the threshold for the significance of genes expression difference. PM and OM: PI 114490 and OH 88119 respectively mock-inoculated with the sterile solution containing 10 mM MgSO_4_·7H_2_O and 0.025% (v/v) Silwet L77. PT and OT: PI 114490 and OH 88119 respectively inoculated with T3.

More stringent criteria with smaller FDR (≤ 0.001) and higher fold-change value (absolute value of Log_2_fold-change ≥ 2) were used to analyze the global transcriptome dynamics of both resistant line PI 114490 and susceptible line OH 88119 in response to race T3 during 6 h and 6 days post-inoculation. Overall, the number of differentially expressed genes (DEGs) was higher in PI 114490 than in OH 88119 at both infection stages, and the number of DEGs was considerably higher at 6 DPI than at 6 HPI in both tomato lines (Figure 1). At the 6 HPI, there were 591 and 299 genes were differentially expressed in PI 114490 and OH 88119, respectively. Most DEGs showed down-regulations in both tomato lines, whereas only 78 and 15 genes were up-regulated in PI 114490 and OH 88119, respectively (Figure 1). However, the numbers of DEGs dramatically increased to 1656 and 1154 at 6 DPI in PI 114490 and OH 88119, respectively, and the numbers of up-regulated genes were obviously greater than that of down-regulated genes (Figure 1). Consideration of numbers and fold changes of these DEGs, resistant tomato line PI 114490 had a higher frequency and stronger fold changes of DEGs at 6 DPI, compared to PI 114490 at 6 HPI and OH 88119 at both time points (Figure 1).

### Genes with differentially expressed patterns in two tomato lines during infection with race T3

**Figure 2:**
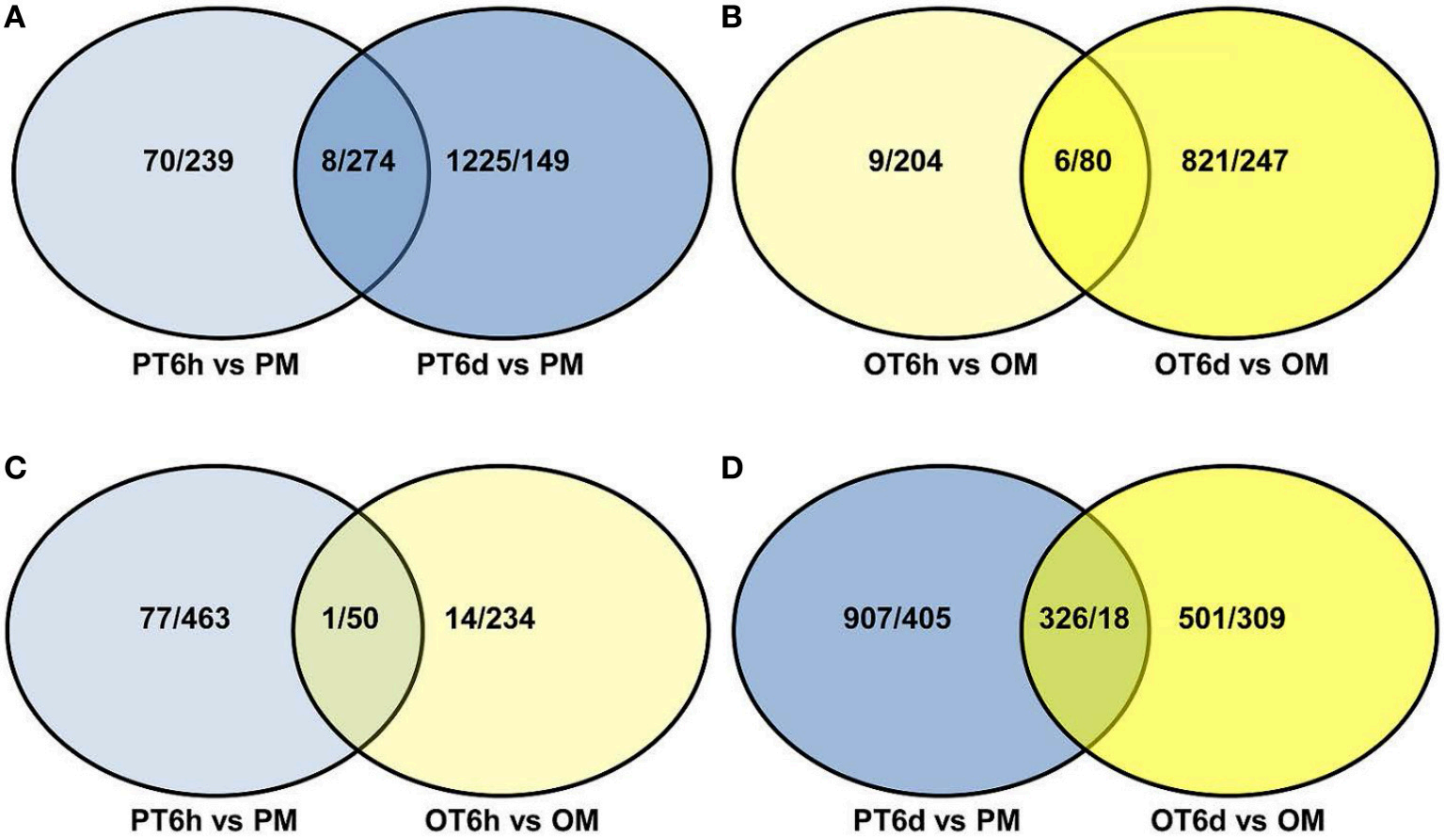
**Venn diagram showing the number of up/down-regulated genes between tomato lines PI 114490 and OH 88119 at 6 h and 6 days post-inoculation with *Xanthomonas perforans* race T3**. PM and OM: PI 114490 and OH 88119 respectively mock-inoculated with the sterile solution containing 10 mM MgSO_4_·7H_2_O and 0.025% (v/v) Silwet L77. PT and OT: PI 114490 and OH 88119 respectively inoculated with race T3. **(A,B)** Comparison of co-DEGs between 6 HPI and 6 DPI in PI 114490 and OH 88119, respectively. **(C,D)** Comparison of co-DEGs between two tomato lines at 6 HPI and 6 DPI, respectively. Overlapping portion indicates co-regulated genes.

In order to gain comprehensive insights into understanding molecular mechanisms of resistance induced by *X. perforans* race T3, the overlap differentially expressed patterns of DEGs between PI 114490 and OH 88119 at two infection stages were analyzed using a Venn diagram (Figure 2). For tomato line PI 114490, 282 genes (8 up-regulated and 274 down-regulated) were co-modulated in both 6 HPI and 6 DPI compared to mock-treatment (Figure 2A), while 86 genes (6 up-regulated and 80 down-regulated) were co-modulated in both 6 HPI and 6 DPI compared to mock-treatment in line OH 88119 (Figure 2B). Comparison between the two tomato lines discovered 51 and 344 DEGs co-regulated at 6 HPI and 6 DPI, respectively (Figures 2C,D). Among the 51 co-regulated DEGs at 6 HPI, 50 were co-down-regulated, and only 1 (Solyc03g114530.2.1) encoding Strictosidine synthase family protein was co-up-regulated. The expression of Strictosidine synthase (Str), the key gene of terpenoid indole alkaloid (TIA) biosynthetic pathway, has been found to be induced by fungal elicitor (Pasquali et al., 1992) and salinity stress in *Catharanthus roseus* (Dutta et al., 2013). Go classification analysis of the 50 down-regulated genes, showed that the dominant subcategories in biological process were response to stimulus (Table S3). Among the 344 DEGs co-regulated at 6 DPI, 326 genes were commonly up-regulated, and 18 genes were commonly down-regulated (Figure 2D). In the face of a large number of commonly up-regulated genes, particular emphasis was placed on the highly up-regulated genes resulting in 32 DEGs with the Log_2_ fold-change ≥ 5 in both lines at 6 DPI (Table 2), of which a large proportion (40.6%) were assigned to Go term “response to stimulus.” For example, marker genes of stress signaling such as pathogen-related genes (PR) and osmotin-like protein were intensely induced, and PR proteins are inducible by infection with various types of pathogens in many plant families (van Loon et al., 2006). In addition to these stimulus genes, other genes also have been clarified related to defense response.

**Table 2:** **RPKM for common highly up-regulated (Log_2_fold-change ≥ 5) genes in tomato lines P I114490 and OH 88119 at 6 days post-inoculation (DPI)**.

**Gene**	**Function description**	**PI 114490**			**OH 88119**		
		**Mock^b^**	**6 DPI^b^**	**Fold value^c^**	**Mock^b^**	**6 DPI^b^**	**Fold-change^c^**
Solyc00g174330.2.1	Pathogenesis related protein^a^	6.37	11.43	5.06	2.88	9.42	6.54
Solyc09g007010.1.1	Pathogenesis related protein^a^	4.69	10.02	5.33	2.63	9.17	6.54
Solyc00g174340.1.1	Pathogenesis-related protein^a^	8.88	14.31	5.43	5.50	12.42	6.93
Solyc01g106620.2.1	Pathogenesis-related protein^a^	4.49	10.20	5.71	1.62	9.27	7.65
Solyc08g080620.1.1	Osmotin-like protein^a^	0	5.38	7.26	0	5.05	6.73
Solyc08g080650.1.1	Osmotin-like protein^a^	6.43	11.47	5.03	4.39	9.75	5.36
Solyc08g080670.1.1	Osmotin-like protein^a^	2.62	7.77	5.15	0	8.14	8.38
Solyc02g082960.2.1	Endochitinase^a^	0	3.30	6.67	0	4.37	7.54
Solyc02g086700.2.1	Beta-1 3-glucanase^a^	0	5.82	7.62	0	6.43	10.04
Solyc01g060020.2.1	Beta-glucanase^a^	4.12	9.30	5.18	0	7.27	7.70
Solyc07g056510.2.1	Glutathione S-transferase^a^	0	5.57	5.46	0	2.11	5.39
Solyc07g066330.2.1	NAC domain protein^a^	0	4.77	6.59	0	1.67	5.28
Solyc01g106630.2.1	Unknown Protein^a^	4.31	9.83	5.51	1.39	8.91	7.53
Solyc12g011150.1.1	Unknown Protein	0	5.52	7.02	0	3.87	5.17
Solyc03g098760.1.1	Kunitz-type protease inhibitor protein^a^	0	2.44	5.16	0	4.70	5.23
Solyc07g052150.2.1	Sesquiterpene synthase	0	6.84	8.65	0	4.83	6.44
Solyc02g093580.2.1	Pectate lyase	0	2.14	6.16	0	1.84	5.67
Solyc01g059990.2.1	Serine/threonine-protein phosphatase 7	0	5.81	5.06	0	3.99	6.36
Solyc02g090970.1.1	Serine/threonine-protein kinase 24	0	3.28	5.83	0	2.93	5.28
Solyc04g074000.2.1	Receptor like kinase, RLK	1.66	6.80	5.14	0	2.19	6.20
Solyc02g071470.2.1	1-aminocyclopropane-1-carboxylate oxidase 1	0	4.71	7.55	0	2.40	6.04
Solyc07g049530.2.1	1-aminocyclopropane-1-carboxylate oxidase	4.43	9.62	5.19	1.97	7.44	5.47
Solyc01g109140.2.1	Cytochrome P450	0	5.41	8.60	0	1.17	5.17
Solyc11g068940.1.1	U-box domain-containing 24	0	3.36	7.14	0	2.17	5.75
Solyc04g008100.1.1	U-box domain-containing	0	3.38	5.22	0	2.46	5.10
Solyc06g062330.1.1	UDP-glucosyltransferase	0	4.63	5.34	0	3.61	6.71
Solyc07g052120.2.1	(−)-germacrene D synthase	0	4.08	6.99	0	3.04	5.75
Solyc07g052140.2.1	(−)-germacrene D synthase	0	7.61	7.71	0	6.45	7.66
Solyc08g029000.2.1	Lipoxygenase	0	6.19	6.70	0	1.96	5.58
Solyc08g068870.2.1	Aspartic proteinase nepenthesin-1	2.28	7.35	5.07	0	5.31	5.38
Solyc12g044950.1.1	Omega-6 fatty acid desaturase	0	2.68	5.04	0	3.46	6.63
Solyc12g049030.1.1	Fatty acid desaturase	0	5.04	5.68	0	3.04	6.49

a *The representative genes are associated with “response to stimulus” according to Gene Ontology classification*.

b *the value of RPKM*.

c *the value of Log_2_(fold-change)*.

**Figure 3:**
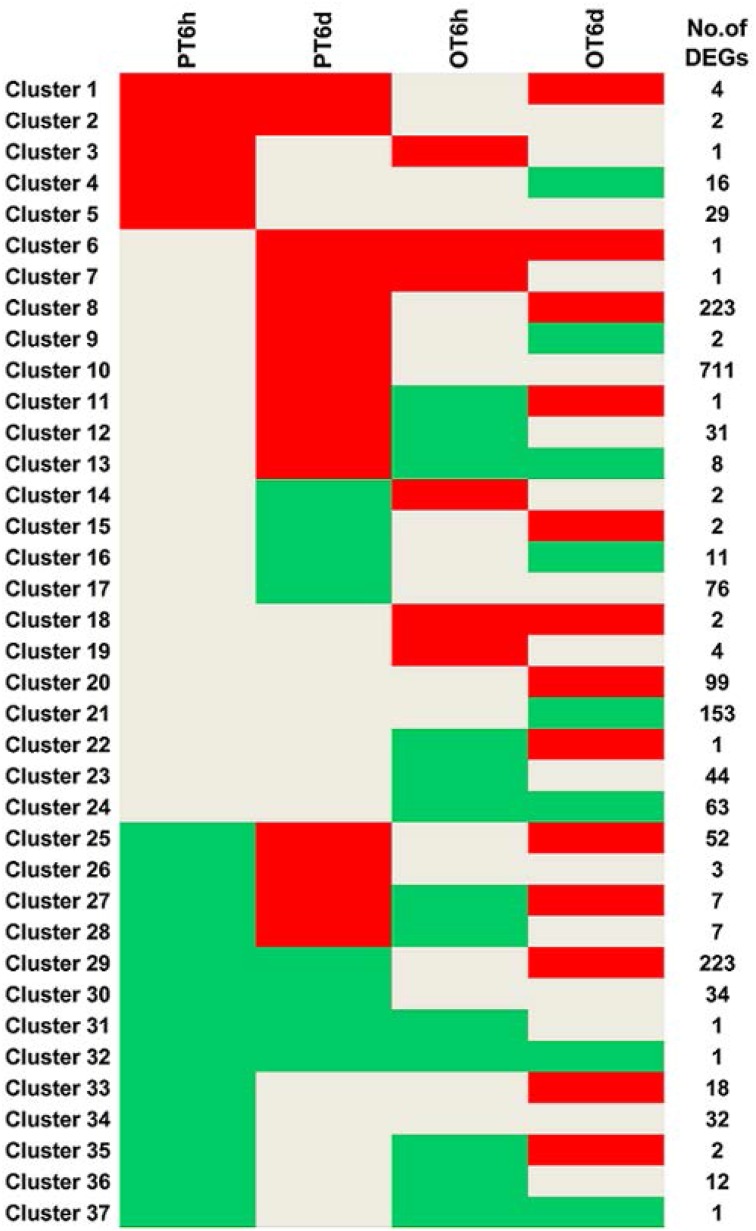
**Hierarchical clustering of differentially expressed genes (DEGs) among four groups**. Up-regulation and down-regulation is represented by red shading and green shading, respectively. Gray shading indicates non-modulated. PT: PI 114490 inoculated with race T3. OT: OH 88119 inoculated with race T3.

Clustering analysis of DEGs among the four comparison groups resulted in 37 clusters (Figure 3). Despite there were substantial number of DEGs, none of these genes was commonly up-regulated at four comparison groups. Cluster 10 was the largest with 711 DEGs, followed by cluster 8 representing 223 common up-regulated genes in PI 114490 and OH 88119 at 6 DPI. Common up-regulated genes in cluster 8 indicated that both PI114490 and OH88119 used some same sets of genes to response to race T3 infection but the induced intensity of transcriptional levels were different between two genotypes. Cluster 10 and cluster 20 consisting of the DEGs were only up-regulated (compared to mock-inoculated) in PI 114490 and OH 88119 at 6 DPI response to race T3. These specific DEGs in PI 114490 from cluster 10 (Table 3) were promising candidates for revealing the resistance response mechanism. Cluster 32 comprised only one unknown function gene Solyc02g084850.2.1 with strongly down-regulated in both PI 114490 and OH 88119 at two infection stages (Table 3). Cluster 27 contained 7 genes strongly down-regulated at 6 HPI but strongly up-regulated at 6 DPI (Table 3), with three genes annotated for pathogenesis-related (Solyc00g174330.2.1, Solyc00g174340.1.1, and Solyc06g009140.2.1). Cluster 13 exhibited that 8 genes were up-regulated in PI 114490 but down-regulated in OH 88119 (Table 3).

**Table 3:** **Part of significantly altered expression genes from Hierarchical clustering analysis**.

**Gene**	**Function description**	**Log**_**2**_**Fold-change value**^**b**^			
		**PI 114490**		**OH 88119**	
		**6 HPI**	**6 DPI**	**6 HPI**	**6 DPI**
**CLUSTER 10 (SPECIFIC UP-REGULATED IN PT6d)**^a^					
Solyc02g087070.2.1	Peroxidase family protein	No	7.6	No	No
Solyc03g020050.2.1	Proteinase inhibitor II	No	8.72	No	No
Solyc06g065060.1.1	FAD-binding domain protein	No	7.4	No	No
Solyc07g008600.1.1	LRR receptor-like serine/threonine-protein kinase	No	7.14	No	No
Solyc07g009500.1.1	Chitinase	No	7.33	No	No
Solyc08g079230.1.1	Cortical cell-delineating protein	No	7.32	No	No
Solyc08g080610.1.1	Osmotin-like protein	No	7.35	No	No
Solyc12g005720.1.1	Cysteine-rich receptor-like kinase	No	7.9	No	No
Solyc12g100250.1.1	Fatty acid desaturase	No	7.6	No	No
**CLUSTER 13 (UP-REGULATED IN PT6d; DOWN-REGULATED IN OT6h AND OT6d)**^a^					
Solyc01g057000.2.1	Universal stress protein	No	6.35	−6.87	−3.96
Solyc03g013440.2.1	Amino acid transporter	No	2.90	−2.91	−2.85
Solyc03g096670.2.1	Integrin-linked kinase-associated serine/threonine phosphatase 2C	No	2.46	−7.00	−4.22
Solyc04g063350.2.1	3-methyl-2-oxobutanoate dehydrogenase	No	2.16	−2.40	−2.33
Solyc06g007180.2.1	Asparagine synthase	No	3.77	−4.52	−2.56
Solyc07g006500.2.1	Alpha alpha-trehalose-phosphate	No	2.52	−2.21	−2.31
Solyc08g077900.2.1	Expansin-like protein	No	3.49	−5.73	−4.06
Solyc12g006230.1.1	RING-H2 finger protein	No	2.05	−4.02	−3.12
**CLUSTER 27 (DOWN-REGULATED IN PT6h AND OT6h; UP-REGULATED IN PT6d AND OT6d)**^a^					
Solyc00g174330.2.1	Pathogenesis related protein PR-1	−8.49	5.06	−5.7	6.54
Solyc00g174340.1.1	Pathogenesis-related protein 1b	−9.1	5.43	−5.41	6.93
Solyc03g025670.2.1	PAR-1c protein	−2.43	4.23	−2.5	2.46
Solyc04g077980.1.1	Zinc-finger protein	−3.75	2.06	−3.2	3.41
Solyc06g009140.2.1	Late embryogenesis abundant 3	−5.78	2.73	−4.74	2.38
Solyc07g049530.2.1	1-aminocyclopropane-1-carboxylate oxidase	−4.14	5.19	−4.54	5.47
Solyc10g085030.1.1	Soul heme-binding family protein	−3.19	2.29	−2.2	2.39
**CLUSTER 32 (DOWN-REGULATED IN PT6h, PT6d, OT6h, AND OT6d)**^a^					
Solyc02g084850.2.1	Unknown Protein	−4.12	−4.86	−12.62	−9.72

a *PT6h: 6 h after inoculation with bacterial spot race T3 in PI 114490. PT6d: 6 day after inoculation with bacterial spot race T3 in PI 114490. OH6h: 6 h after inoculation with bacterial spot race T3 in OH 88119. OH6d: 6 day after inoculation with bacterial spot race T3 in OH 88119*.

b *“NO” indicates that the expression level of the genes after infection with race T3 is not significant difference as relative to that of mock-treatment*.

### Gene ontology classification analysis of DEGs

Go enrichment categorized for 52.5, 54.2, 50.5, and 51.2% of DEGs into functional groups from PT6h, OT6h, PT6d, and OT6d, respectively. More assigned function of DEGs was covered in biological process and molecular function categories than in cellular component categories (Image S2). The dominant subcategories of both PI 114490 and OH 88119 at 6 HPI and 6 DPI in each main category were cellular process, cell and catalytic activity, respectively (Image S2). Despite these enrichment subcategories were similar in both tomato lines, the individual genes contributing to the common enriched subcategories were substantial diversified between the two tomato lines.

The most significantly enriched GO terms in biological process in the OT6d library included “response to wounding (Go:0009611),” “response to external stimulus (GO:0009605),” “oxidation reduction (GO:0055114),” “response to stress (GO:0006950),” and “response to biotic stimulus (GO:0009607),” For the library of PT6d, the most significantly enriched GO terms were “cell wall macromolecule metabolic process (GO:0044036)” and “response to water (GO:0009415)” (Table S4).

### Metabolic pathway by KEGG analysis of DEGs

To characterize the pathway enrichment of the identified DEGs, gene classification was performed on the basis of KEGG analysis (Kanehisa et al., 2010). Only significant pathway categories among four comparisons were selected and listed in Table 4. Genes involved in photosynthesis and oxidative phosphorylation pathways were mainly up-regulated in OH 88119 at 6 DPI, but down-regulated in PI114490 at 6 HPI and 6 DPI (Table 4). It was obvious that genes associated with defense response pathways (labeled in bold in Table 4) were significantly up-regulated in PI114490 at 6 DPI. The defense-related pathways with most representation by DEGs were plant hormone signal transduction, plant-pathogen interaction and phenylalanine metabolism. A total of 16 defense response genes in KEGG pathway “plant-pathogen interaction” (KO: 04626) exhibited significant differences between PI 114490 and OH 88119 in response to T3 infection (Table 5). Of which, 5 and 3 were down-regulated at 6 HPI in PI 114490 and OH 88119, respectively. However, the numbers of DEGs increased in both tomato lines at 6 DPI. A total of 15 (14 up-regulated and 1 down-regulated) in PI 114490 and 8 (6 up-regulated and 2 down-regulated) gens in OH 88119 was detected. It was noteworthy that 5 DEGs (Solyc05g050350.1.1, Solyc10g079420.1.1, Solyc11g071740.1.1, Solyc09g014990.2.1, and Solyc12g009220.1.1) had the similar expression pattern between two tomato lines at both 6 HPI and 6 DPI (Table 5).

**Table 4:** **Significantly enriched KEGG pathways of differentially expressed genes by *Xanthomonas perforans* race T3 infection**.

	**Pathway**	**No. of up-regulated genes**	**No. of down-regulated genes**	**Pathway ID**
PT6h vs. PM	Photosynthesis	0	23	Ko 00195
	Oxidative phosphorylation	0	27	Ko 00190
	Ribosome	0	13	Ko 03010
PT6d vs. PM	Photosynthesis	0	23	Ko 00195
	Oxidative phosphorylation	1	28	Ko 00190
	Phenylalanine metabolism	9	0	Ko 00360
	Glutathione metabolism	10	1	Ko 00480
	Phenylpropanoid biosynthesis	11	2	Ko 00940
	Ribosome	0	14	Ko 03010
	Plant hormone signal transduction	15	3	Ko 04075
	Plant-pathogen interaction	14	1	Ko 04626
OT6h vs. OM	Protein processing in endoplasmic reticulum	0	10	Ko 04141
	MAPK signaling pathway	0	4	Ko 04010
	Plant hormone signal transduction	0	9	Ko 04075
OT6d vs. OM	Oxidative phosphorylation	25	0	Ko 00190
	Photosynthesis	19	1	Ko 00195
	Ribosome	11	1	Ko 03010
	Phenylalanine metabolism	7	1	Ko 00360
	Metabolism of xenobiotics by cytochrome P450	9	0	Ko 00980

*PM and OM: PI 114490 and OH 88119 respective mock-treatment with the sterile solution containing 10 mM MgSO_4_.7H_2_O and 0.025%(v/v) Silwet L77. PT and OT: inoculation with bacterial spot race T3 in PI 114490 and OH 99119, respectively*.

**Table 5:** **Differentially expressed genes in the common enriched KEGG pathway “Plant-pathogen interaction”(ko 04626) in tomato lines PI 114490 and OH 88119 at 6 h post-inoculation (HPI) and 6 days post-inoculation (DPI)**.

**Gene**	**Homologous protein in KEGG**	**PI 11449**					**OH 88119**				
		**RPKM**			**Log**_**2**_**Fold-change**^**a**^		**RPKM**			**Log**_**2**_**Fold-change**^**a**^	
		**Mock**	**6 HPI**	**6 DPI**	**6 HPI vs. Mock**	**6 DPI vs. Mock**	**Mock**	**6 HPI**	**6 DPI**	**6 HPI vs. Mock**	**6 DPI vs. Mock**
Solyc03g113390.2.1	CPK	7.25	7.21	38.92	−0.008	**2.42**	8.62	6.89	16.01	−0.32	0.89
Solyc06g065380.2.1	CPK	3.06	4.83	0.68	0.66	−**2.15**	3.17	5.97	1.86	0.91	−0.76
Solyc05g050350.1.1	CNGF	0.25	0	2.74	0	**3.41**	0.09	0.11	0.88	0.26	**3.16**
Solyc10g081170.1.1	CALM	336.89	336.1	1391.1	−0.003	**2.05**	189.18	336.69	387.5^a^	0.83	1.03
Solyc03g118810.1.1	CML	83.01	14.98	235.87	−**2.46**	1.51	14.15	8.74	96.89^a^	−0.69	**2.77**
Solyc06g068960.1.1	CML	31.84	30.13	161.84	−0.07	**2.35**	13.69	18.07	21.85	0.39	0.67
Solyc10g079420.1.1	CML	41.93	25.34	257.87	−0.72	**2.62**	19.58	23.48	131.57^a^	0.26	**2.75**
Solyc02g094000.1.1	CML	3.04	0	32.47	−**3.65**	**3.42**	0.49	0	11.18^a^	0	**4.49**
Solyc03g044900.2.1	CML	5.19	6.71	25.07	0.36	**2.27**	8.34	10.58	6.06	0.34	−0.46
Solyc06g073830.1.1	CML	0.66	0	19.27	0	**4.85**	1.147	0	1.71	0	0.58
Solyc11g071740.1.1	CML	5.19	0.34	74.57	−**3.93**	**3.84**	4.20	10.42	18.34	−**3.32**	**2.13**
Solyc11g071760.1.1	CML	0	0	8.34	0	**5.75**	1.42	0	2.66	0	0.90
Solyc09g014990.2.1	WRKY33	2.52	0.11	28.48	−**4.43**	**3.50**	0.84	0.14	12.26	−**2.54**	**3.86**
Solyc06g036290.2.1	HSP90	16.45	2.76	70.45	−**2.57**	**2.09**	32.76	2.02	18.11	−**4.0**	−0.85
Solyc07g042170.2.1	JAZ	15.32	10.98	69.14	−0.48	**2.17**	15.35	13.32	60.44	−0.20	1.98
Solyc12g009220.1.1	JAZ	3.41	7.33	75.82	1.10	**4.47**	6.43	7.71	33.47	0.26	**2.38**

a *Significant difference (FDR ≤ 0.001 and the absolute value of Log_2_ fold-change ≥ 2) were shown in boldface*.

### Validation of RNA-seq data for selected genes by RT-PCR and qRT-PCR

**Figure 4:**
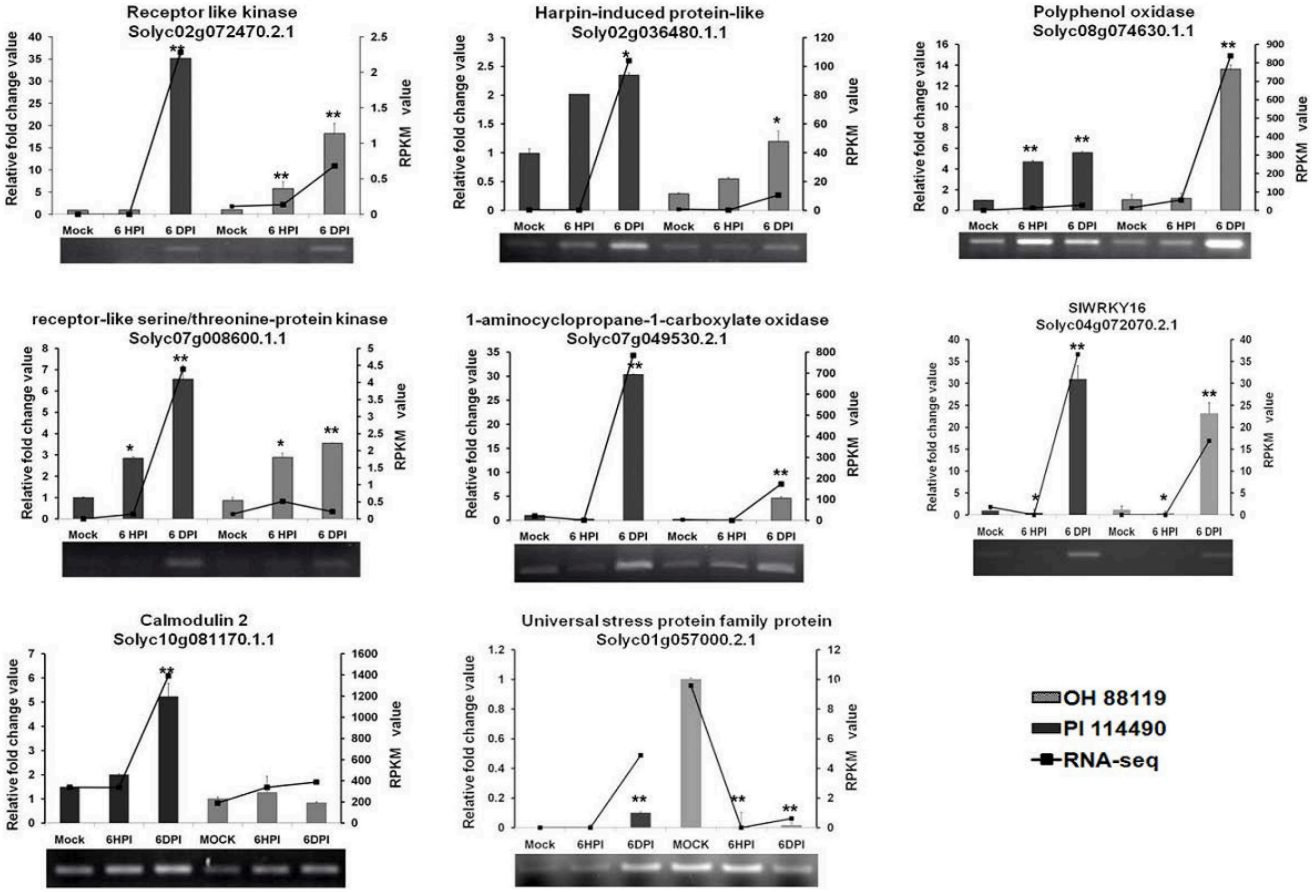
**RT-PCR and qRT-PCR validation of differentially expressed 8 genes related to defense response at 6 h post-inoculation (6 HPI) and 6 days post-inoculation (6 DPI)**. Expression levels of each sample were normalized on the basis of transcript amounts of *EF1*-α. Relative expression values for qRT-PCR analysis were determined against the average value of mock sample with PI 114490. Each experiment determined in three technical replicates and standard deviation (*n* = 3) is represented by error bars. The asterisk above the bars indicates statistically significant differences between the infected samples and corresponding mock samples.

To evaluate the validity of RNA-seq analysis, transcriptional levels of selected 8 DEGs representing a wide range of expression levels and patterns were determined by RT-PCR and qRT-PCR analysis. Based on the functional annotation in literatures, majority of these DEGs were associated with massive defense response processes including ethylene biosynthesis (Solyc07g049530.2.1), defense-related enzymes (Solyc02g072470.2.1, Solyc07g008600.1.1, Solyc01g057000.2.1, Solyc02g036480.1.1, Solyc04g072070.2.1 and Solyc10g081170.1.1), and second metabolite biosynthesis (Solyc08g074630.1.1). In general, the expression data provided by qRT-PCR were almost consistent with profiles detected by RNA-seq at all time-points (Figure 4), confirming the trends of up- or down-regulation of all the analyzed genes. The differences in the magnitudes of changes observed between the qRT-PCR and RNA-seq results might be caused by different algorithms.

It was validated by qRT-PCR that four genes Solyc02g072470.2.1, Solyc02g036480.1.1, Solyc08g074630.1.1, and Solyc07g008600.1.1 were steadily up-regulated during two time-points infection in both PI 114490 and OH 88119, differing in their induced strength. Solyc07g049530.2.1 and Solyc04g072070.2.1 initially displayed down-regulated at 6 HPI but significantly reinforced at 6DPI in both tomato lines, and the up-regulated fold-change was higher in PI 114490. The expression pattern of Solyc01g057000.2.1 was opposite between two tomato lines, which was up-regulated in the resistant line PI 114490 while down-regulated in the susceptible line OH 88119 (Figure 4).

## Disscussion

The *S. lycopersicum* var. *cerasiforme* accession PI 114490 has been considered as a durable source for resistance to bacterial spot due to its high level of quantitative field resistance to four races T1–T4 (Yang, 2013). There is no or few lesions on PI 114490, but the bacterial population of race T3 in its leaves is not significantly different from that in the susceptible line OH 88119 (Sun et al., 2014). So considering on the aspect of phenotype, PI 114490 shows higher tolerant response to avoid the development of symptoms. But the difference of global inducible defense response against *X. perforance* race T3 infection between PI 114490 and OH 88119 were not clear.

In this study, RNA-seq technique was adapted to detect the DEGs during the race T3 infection in PI 114490 and OH 88119. Our previous study using cDNA-AFLP techniques has already identified DEGs between the two tomato lines (Du et al., 2014). Therefore, we started with one biological replication for RNA-seq analysis. An average of 7 million sequence reads were obtained from RNA-seq and the clean reads could match approximately 62% of reference genes (Table 1), which was closed to previous tomato RNA-seq data with 20 million sequence reads (Tang et al., 2013), indicating that the sequencing depth was sufficient for the transcriptome coverage. The well correlation between qRT-PCR and RNA-seq data (Figure 4) also demonstrated the reliability of RNA-seq analysis. Of course, more replicates should provide more reliable information for understanding the genes involved in the process of resistance/defense to the pathogen of bacterial spot race T3 in tomato.

### Inducible defense response to *X. perforans* interfered at 6 HPI but activated at 6 DPI in both resistant and susceptible tomato lines

Despite PI 114490 and OH 88119 have different responses to race T3 infection (Sun et al., 2014), lots of common DEGs were regulated in both two tomato lines. At 6 HPI, 50 common down-regulated genes existed in both two tomato lines (Figure 2C) suggested that the inducible defense response pathways were not completely activated at 6 HPI. Meanwhile, down-regulation of the minority of pathogen-related genes in cluster 27 (Figure 3) indicated that at least some defense response had been interfered by pathogen infection as early as 6 HPI.

Among the 344 common DEGs at 6 DPI, a majority of 326 genes were commonly up-regulated (Figure 2D) in both tomato lines. The dominant subcategories in biological process were cellular process, macromolecule metabolism and response to stimulus (data not shown). Six up-regulated genes in plant-pathogen interaction pathway were shared by the two tomato lines at 6 DPI (Table 5). Previous studies have proven that the function of these regulated genes plays important role in bacterial spot defense response (Cui et al., 2015). Therefore, defense response of tomato lines was activated 6 days after infection by race T3. Accumulation expression levels of those co-regulated genes were likely to increase basal defense response and improve the disease resistance to race T3 infection in both resistant and susceptible tomato. So the susceptible line OH 88119 also succeeded to properly activate the expression of many defense-related genes.

### Resistant and susceptible tomato lines displayed differentially expressed patterns in photosynthesis pathway

Plant-pathogen interaction response usually alters the expressional level of genes associated with photosynthesis (Major et al., 2010). Many photosynthesis-related genes including chlorophyII a/b binding proteins and photosynthetic reaction center proteins were strongly repressed in susceptible hybrid poplar leaves at 9 days inoculated with *Melampsora medusa* (Miranda et al., 2007). However, our RNA-seq data showed that race T3 pathogen caused opposite impact on photosynthesis between resistance and susceptible tomato lines. Genes involved in photosynthesis pathway were mainly up-regulated in the susceptible tomato line OH 88119 at 6 DPI, but down-regulated in the resistant line PI 114490 at 6 HPI and 6 DPI (Table 4). To the best of our knowledge, the reason of this divergent response between resistant and susceptible plants in photosynthesis pathway has not previously been known in plant-pathogen interactions.

### Analysis of differentially expressed genes involved in plant immune response pathways

Plants possess different sophisticated defense strategies which are usually accompanied by transcriptional changes to protect themselves against pathogen attacks. The plant immune response pathway can be mainly divided into two branches (Jones and Dangl, 2006). The first branch and initial defense response is usually activated by pathogen-associated molecular patterns (PAMP) whose presence is recognized by plant plasma membrane-localized pattern recognition receptors (PRRs), which results in PAMP-triggered immunity (PTI). The second branch is activated by nucleotide-binding leucine-rich repeat (NB-LRR) resistance gene, which directly or indirectly interacts with virulence factor, and results in activation of effector-triggered immunity (ETI). Since the plant-pathogen interaction pathways (KEGG: 04626) summarized the genes involved in defense network of PTI and ETI, so we focused on the transcript dynamics of this KEGG pathways and enumerated the DEGs imparting the plant immune response (Table 5).

Several PAMPs have been identified from bacteria. Flagellin is one of important and well-characterized PAMPs that can be recognized by the LRR receptor-like serine/threonine-protein kinase (FLS2) (Gómez-Gómez and Boller, 2000). In our study, RLK protein Solyc02g070890.2.1 with the highest amino acid similarity (54%) to FLS2 protein from *Arabidopsis thaliana* than other tomato RLK genes was down-regulated at 6 HPI, and slightly up-regulated at 6 DPI in PI 114490, but this gene was not differentially expressed in OH 88119 upon race T3 infection. RLK protein Solyc02g072470.2.1 was the highest induced member of RLK family in our RNA-seq data. qRT-PCR validated that Solyc02g072470.2.1 were significantly induced in both tomato lines at 6 DPI, which displayed more than 35- and 18-fold-induction in PI 114490 and OH 88119, respectively (Figure 4). The highest induced member of LRR receptor-like serine/threonine-protein kinase was Solyc07g008600.1.1 in RNA-seq analysis, which was verified up-regulated in both two tomato lines (Figure 4).

**Figure 5:**
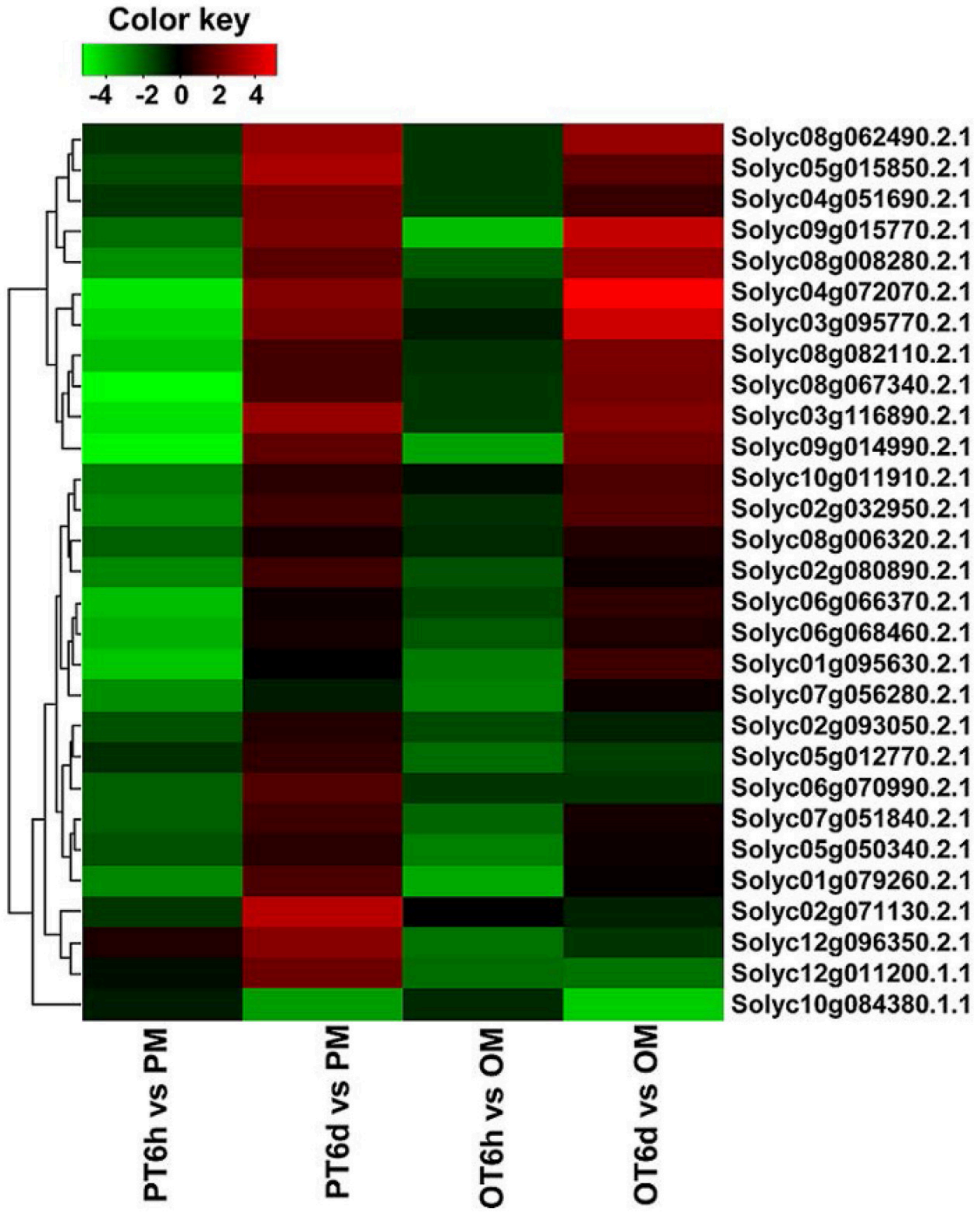
**Differentially expressed WRKY genes in tomato lines PI 1144490 and OH 88119 at 6 h and 6 days post-inoculation with *Xanthomonas perforans* race T3**. PM and OM: PI 114490 and OH 88119 respectively mock-inoculated with the sterile solution containing 10 mM MgSO_4_·7H_2_O and 0.025% (v/v) Silwet L77. PT and OT: PI 114490 and OH 88119 respectively inoculated with race T3.

Recognition of PAMPs initiates downstream signaling pathways involving WRKY transcription factors to confer defense response against bacterial, fungal pathogens and nematodes (Asai et al., 2002; Bhattarai et al., 2010). Based on KEGG analysis, WRKY transcription factor Solyc09g014990.2.1 homologous to WRKY33 was down-regulated at 6 HPI but highly up-regulated at 6 DPI in both genotypes (Table 5). Since members of WRKY families are thought to play important role in defense response, we further investigated the expression patterns of all 85 WRKY families genes annotated in the International Tomato Annotation Group (ITAG) Release 2.3 Predicted CDS. A total of 29 WRKY genes were differentially expressed. Among them, none was significantly up-regulated at 6 HPI but 17 (58.6%) were up-regulated in both tomato lines at 6 DPI (Figure 5). Previous study indicated that the expression levels of SlWRKY8 (Solyc02g093050.2.1), SlWRKY23 (Solyc01g079260.2.1), SlWRKY39 (Solyc03g116890.2.1), SlWRKY53 (Solyc08g008280.2.1), SlWRKY80 (Solyc03g095770.2.1), and SlWRKY81 (Solyc09g015770.2.1) were enhanced in *S. lycopersicum* cultivar Alisa Craig under the invasion of *Pseudomonas syringae* (Huang et al., 2012). In the current study, none of these WRKY genes were detected at 6 HPI, but SlWRKY39/53/80/81 were significantly induced in both tomato lines, and SlWRKY8/23 were specific up-regulated in PI 114490 at 6 DPI. SlWRKY72b are transcriptionally up-regulated during disease resistance mediated by the R gene *Mi-1*, and virus-induced gene silencing of this gene in tomato resulted in a clear reduction of *Mi-1*-mediated resistance as well as basal defense against root-knot nematodes and potato aphids (Bhattarai et al., 2010). WRKY gene Solyc06g070990.2.1, the highest identity with SlWRKY72b than other WRKY protein in tomato, were significantly induced at 6 DPI in PI 114490 (Figure 5).

Intracellular Ca^2+^ influxes have also long been recognized as essential and early events downstream of multiple PAMP perception, leading to local and systemic acquired resistance (Lecourieux et al., 2005; Boudsocq et al., 2010). In the process of Ca^2+^ signaling, some members of Calcium-dependent protein kinase (CDPK) family was immediately induced by flg22 and Avr-Cf9 interaction (Romeis et al., 2000; Boudsocq et al., 2010). Unexpectedly, none of CDPK genes was induced as early as 6 HPI in both tomato lines. Calmodulin (CALM or CaM), a major Ca^2+^ sensor, play positive regulatory resistance roles in different host-pathogen interaction (Park et al., 2004; Choi et al., 2009). Only one CALM gene Solyc10g081170.1.1 was specifically up-regulated at 6 DPI in PI 114490 (Table 5), which was also validated by qRT-PCR analysis (Figure 4).

**Figure 6:**
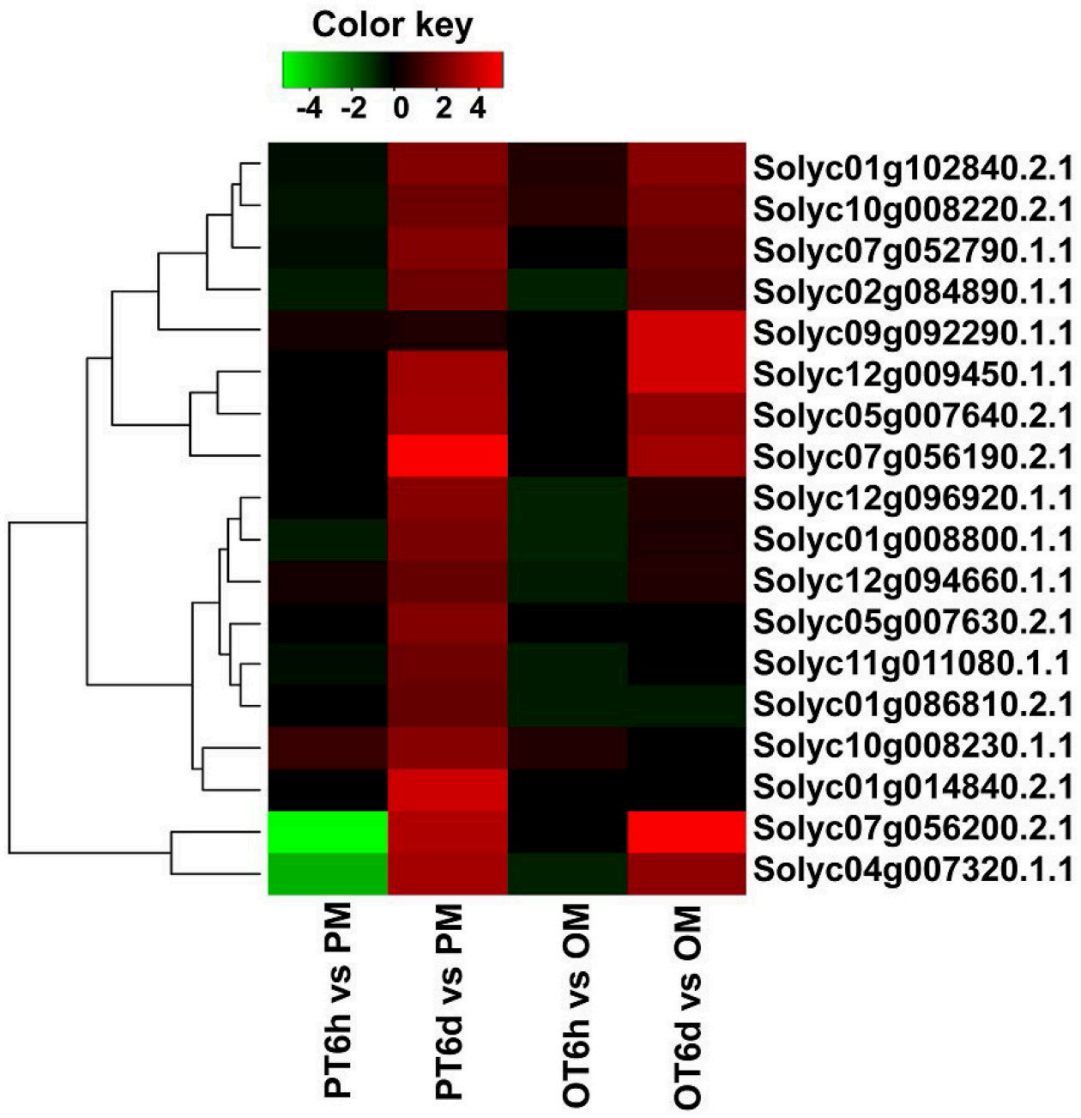
**Differentially expressed putative R genes containing NBS-LRR domain in tomato lines PI 1144490 and OH 88119 at 6 h and 6 days post-inoculation**. PM and OM: PI 114490 and OH 88119 respectively mock-inoculated with the sterile solution containing 10 mM MgSO_4_·7H_2_O and 0.025% (v/v) Silwet L77. PT and OT: PI 114490 and OH 88119 respectively inoculated with race T3.

In the process of plant and pathogen interaction, bacterial type III effectors which are directly delivered into the host cells via the type III secretion system can bypass or disrupt host first line of PAMP-triggered immunity. But resistant plants contain R proteins belonging to the nucleotide -binding site-leucine-rich repeat (NBS-LRR) family to directly or indirectly detect bacterial effectors, which will activate downstream signaling and lead to pathogen resistance (DeYoung and Innes, 2006). Previous papers have evidence that some R genes can be activated by the specific effectors, which are displayed by the accumulation of R genes expression levels. Therefore, we paid attention to those putative R genes containing NBS-LRR domain that could possibly be induced by the *X. perforans* infection. A total of 298 genes were annotated as NBS-LRR proteins for tomato in SGN database, and the RPKM values of most genes were low in the six sequenced samples, indicating the expression level of most putative R genes was low in tomato plant. The RNA-seq data showed that none of putative R genes were up-regulated in both tomato lines at 6 HPI. But the number of up-regulated putative R genes in PI 114490 at 6 DPI was more than that of in OH 881119 (Figure 6). Six putative R genes were commonly up-regulated in both tomato lines. Among these genes, Solyc07g056190.2.1 displayed the highest fold-change (Figure 6). In addition, there were 11 and 1 putative R genes were specifically up-regulated in P I114490 and OH88119, respectively (Figure 6).

### Enumerate of significant *X. perforans* up-regulated genes

Previously, we used cDNA-AFLP technique to identify differential expression genes in tomato lines PI 114490 and OH 88119 in response to race T3 infection at 3, 4, and 5 days. A total of 60 genes were commonly up-regulated at different levels in two tomato lines (Du et al., 2014). Among these genes, 14 genes were also up-regulated in this study (Table S5). Based on the functional annotation in literatures, majority of these genes were associated with massive defense response process including jasmonic acid biosynthesis pathways (Solyc03g122190.2.1 and Solyc04g079730.1.1), ethylene biosynthesis (Solyc07g049530.2.1), defense-related enzymes (Solyc10g055800.1.1, Solyc00g071180.2.1, Solyc03g007240.2.1, and Solyc04g015970.2.1), and cell death related (Solyc03g006700.2.1, Solyc01g105070.2.1, Solyc08g074680.2.1, and Solyc11g068940.1.1). The remaining (Solyc05g050120.2.1, Solyc08g068710.1.1, and Solyc04g010250.2.1) did not match any known defense response genes. Similar expression pattern in different expression profile techniques indicates that these well-validated and reproducible 14 up-regulated genes may play important role in tomato-*X. perforans* interaction.

## Conclusion

In this study, high-throughput sequencing data was used to identify *X. perforans* responsive modules, which were enriched for differentially expressed genes between resistant line PI 114490 and susceptible line OH 88119 at different time point after spray-inoculation of race T3. The data provided a whole view on the variation tendency of genes involved in tomato-*X. perforans* interaction. A large number of candidate genes involved in tomato-*X. perforans* interaction were identified. Comparison of RNA-seq data with our previous cDNA-AFLP data revealed common up-regulated genes, and some of them were involved in jasmonic acid and ethylene biosynthesis.

## Author contributions

Conceived and designed the experiments: YW, WY. Performed the experiments: HD. Analyzed the data: HD, JY, WY. Contributed reagents/materials/analysis tools: YW, JY, WY. Wrote the paper: HD, WY.

### Conflict of interest statement

The authors declare that the research was conducted in the absence of any commercial or financial relationships that could be construed as a potential conflict of interest.

## Supplementary material

Table S1 **Genes and oligonucleotide sets used in quantitative and semi-quantitative RT-PCR experiments**.

Table S2 **Statistics of total tomato reference genes in different expression level intervals**.

Table S3 **Common down-regulated genes associated with “response to stimulus” according to Gene Ontology classification in tomato lines PI 114490 and OH 88119 at 6 h post-inoculation (HPI)**.

Table S4 **Gene ontology (GO) analysis of up-regulated genes for biological process in tomato lines PI 114490 and OH 88119 at 6 d post-inoculation**.

Table S5 **Sets of common differentially expressed genes in RNA-seq analysis with previous cDNA-AFLP results**.

Image S1 **Venn diagram showing the overlaps and specific expressed genes between mock samples and samples spray-inoculated with *Xanthomonas perforans* race T3 in tomato lines PI 114490 and OH 88119**. PM and OM: PI 114490 and OH 88119 respective mock-treatment with the sterile solution containing 10 mM MgSO_4_.7H_2_O and 0.025%(v/v) Silwet L77. PT and OT: inoculation with bacterial spot race T3 in PI 114490 and OH 88119, respectively.

Image S2 **Distribution of the differentially expressed genes (DEGs) within Go secondary categories of molecular function, cellular component and biological process. (A)** Go classification for DEGs identified from OT6h vs. OM. **(B)** Go classification for DEGs identified from OT6d vs. OM. **(C)** Go classification for DEGs identified from PT6h vs. PM. **(D)** Go classification for DEGs identified from PT6d vs. PM. PM and OM: PI 114490 and OH 88119 respective mock-treatment with the sterile solution containing 10 mM MgSO_4_.7H_2_O and 0.025%(v/v) Silwet L77. PT and OT: inoculation with bacterial spot race T3 in PI 114490 and OH 88119, respectively.
