# Supplementary material for: Comparative Transcriptome Analysis of Resistant and Susceptible Tomato Lines in Response to Infection by Xanthomonas perforans Race T3
Source: Front Plant Sci. 2015 Dec 24;6:1173. doi: 10.3389/fpls.2015.01173 (PMC4689867; doi:10.3389/fpls.2015.01173)
Supplement: Table S1 — Genes and oligonucleotide sets used in quantitative and semi-quantitative RT-PCR experiments. [file Table1.DOCX]

**Table S1** Genes and oligonucleotide sets used in quantitative and semi-quantitative RT-PCR experiments.

| **Gene** | **Gene Function** | **Primers (5’ to 3’)** |
| --- | --- | --- |
| Solyc02g072470.2.1 | Receptor like kinase | F: ATTGCACCAGAGTATGGAAG  R: CCAAACACTTTCTATACCCTCAA |
| Solyc08g074630.1.1 | Polyphenol oxidase | F: AATCATCGCAATGGAACTA  R: ACAATCACGGACTTTCACG |
| Solyc02g036480.1.1 | Harpin-induced protein-like | F: AACCTCAACACCGATACGA  R: AGGCTGCACTTCAACAAGA |
| Solyc07g008600.1.1 | LRR receptor-like serine/threonine-protein kinase | F: CTGAATCTTCGTGGTAACTTC  R: TTGGTATTTGTCCACCAAAA |
| Solyc07g049530.2.1 | 1-aminocyclopropane-1-carboxylate oxidase | F: GGGACATTACAAGAAGTGCA  R: GCTTAGGACATGGTGGATAG |
| [Solyc01g057000.2.1](http://solgenomics.net/feature/17676971/details) | Universal stress protein family protein | F: CTTTCTTGGGTAGTGTGAGC  R: AAAACCTATTGCCTGTGGAC |
| Solyc04g072070.2.1 | WRKY transcription factor 16 | F: GATGGATACAAATGGAGGAA  R: CCAGTAAATGATAAAGGGAC |
| [Solyc10g081170.1.1](http://solgenomics.net/feature/17961802/details) | Calmodulin-2 | F: CTGATGAAGAAGTCGATGAGATG  R: AGACAAGAGCCTACCCAATGA |
| *EF1*-α |  | F: TACTGGTGGTTTTGAAGCTG  R: AACTTCCTTCACGATTTCATCATA |
|  |  |  |
