# Supplementary material for: Comparative Transcriptome Analysis of Resistant and Susceptible Tomato Lines in Response to Infection by Xanthomonas perforans Race T3
Source: Front Plant Sci. 2015 Dec 24;6:1173. doi: 10.3389/fpls.2015.01173 (PMC4689867; doi:10.3389/fpls.2015.01173)
Supplement: Table S2 — Statistics of total tomato reference genes in different expression level intervals. [file Table2.DOCX]

**Table S2** Statistics of total tomato reference genes in different expression level intervals.

| RPKM interval |  | Number of genes^a^ | | | | | |
| --- | --- | --- | --- | --- | --- | --- | --- |
|  | PM | PT6h | PT6d | OM | OT6h | OT6d | Average(%)^b^ |
| = 0 | 12,951 | 13,382 | 12,734 | 13,019 | 13,559 | 13,559 | 13,200.6(38.01) |
| 0-0.1 | 199 | 143 | 171 | 111 | 83 | 29 | 122.7(0.35) |
| 0.1-3 | 7,494 | 7,241 | 6,385 | 6,452 | 6,528 | 6,472 | 6,762(19.47) |
| 3-10 | 6,315 | 6,291 | 5,387 | 6,040 | 6,063 | 6,183 | 6,046.5(17.41) |
| 10-40 | 5,344 | 5,289 | 6,563 | 6,323 | 5,827 | 5,727 | 5,845.5(16.83) |
| >40 | 2,424 | 2,381 | 3,487 | 2,782 | 2,667 | 2,757 | 2,749.7(7.92) |

**^a^** PM and OM: PI 114490 and OH88119 respective mock-inoculated with the sterile solution containing 10 mM MgSO_4_·7H_2_O and 0.025%（v/v）Silwet L77.PT and OT: PI 114490 and OH 88119 respectively inoculated with T3.

**^b^**The numbers in parentheses are the percentage of genes based on the number of tomato 34,727 reference genes.
