# Supplementary material for: Comparative Transcriptome Analysis of Resistant and Susceptible Tomato Lines in Response to Infection by Xanthomonas perforans Race T3
Source: Front Plant Sci. 2015 Dec 24;6:1173. doi: 10.3389/fpls.2015.01173 (PMC4689867; doi:10.3389/fpls.2015.01173)
Supplement: Table S3 — Common down-regulated genes associated with “response to stimulus” according to Gene Ontology classification in tomato lines PI 114490 and OH 88119 at 6 h post-inoculation (HPI). [file Table3.DOCX]

**Table S3** Common down-regulated genes associated with “response to stimulus” according to Gene Ontology classification in tomato lines PI 114490 and OH 88119 at 6 h post inoculation (HPI).

| **Gene** | **Function description** | **PI 114490** | | |  | **OH 88119** | | |
| --- | --- | --- | --- | --- | --- | --- | --- | --- |
|  |  | **Mock^a^** | **6 HPI^a^** | **Fold value^b^** |  | **Mock^a^** | **6 HPI^a^** | **Fold-change^b^** |
| Solyc00g174340.1.1 | Pathogenesis-related protein 1b | 470.48 | 0.85 | -9.09 |  | 45.11 | 1.06 | -5.41 |
| Solyc01g109250.2.1 | TMV response-related protein | 9.50 | 1.176 | -3.01 |  | 8.23 | 1.45 | -2.50 |
| Solyc02g062960.2.1 | Homeobox-leucine zipper protein | 4.46 | 0.64 | -2.78 |  | 17.35 | 0.53 | -5.02 |
| Solyc02g089540.2.1 | Tomato CONSTANS-like 1 | 18.15 | 2.04 | -3.14 |  | 17.13 | 1.26 | -3.75 |
| Solyc03g093560.1.1 | Ethylene-responsive transcription factor 2 | 41.05 | 1.40 | -4.87 |  | 12.13 | 1.03 | -3.54 |
| Solyc03g119530.2.1 | LOB domain protein 42 | 20.36 | 3.77 | -2.43 |  | 17.41 | 3.93 | -2.14 |
| Solyc03g121090.2.1 | Cold induced protein-like | 31.56 | 3.98 | -2.98 |  | 52.11 | 11.39 | -2.19 |
| Solyc04g005610.2.1 | NAC domain transcription factor | 9.34 | 0.96 | -3.27 |  | 16.66 | 0 | -7.12 |
| Solyc04g011440.2.1 | heat shock protein | 5.02 | 0.67 | -2.88 |  | 21.02 | 0.73 | -4.84 |
| Solyc04g079420.2.1 | Nbs-lrr, resistance protein | 36.98 | 3.66 | -3.33 |  | 13.37 | 2.26 | -2.56 |
| Solyc06g036290.2.1 | Heat shock protein 90 | 16.45 | 2.76 | -2.57 |  | 32.75 | 2.02 | -4.01 |
| Solyc07g006370.1.1 | Sodium/calcium exchanger protein | 14.12 | 3.26 | -2.11 |  | 12.70 | 2.98 | -2.08 |
| Solyc07g049530.2.1 | 1-aminocyclopropane-1-carboxylate oxidase | 21.56 | 1.22 | -4.142 |  | 3.91 | 0.16 | -4.54 |
| Solyc07g053740.1.1 | Ethylene-responsive transcription factor 4 | 189.75 | 21.27 | -3.15 |  | 60.43 | 9.76 | -2.62 |
| Solyc07g063120.2.1 | WD-40 repeat protein | 5.57 | 1.21 | -2.20 |  | 9.25 | 1.05 | -3.12 |
| Solyc09g089730.2.1 | 1-aminocyclopropane-1-carboxylate oxidase-like protein | 52.88 | 10.95 | -2.2 |  | 100.80 | 14.05 | -2.84 |
| Solyc09g089930.1.1 | Ethylene responsive transcription factor 1a | 88.84 | 0.57 | -7.28 |  | 18.20 | 1.76 | -3.36 |
| Solyc10g005080.2.1 | Late elongated hypocotyl and circadian clock associated-1-like | 9.21 | 1.25 | -2.87 |  | 16.67 | 1.46 | -3.50 |
| Solyc10g009110.1.1 | Ethylene-responsive transcription factor 4 | 92.69 | 3.38 | -4.77 |  | 18.08 | 1.90 | -3.24 |
| Solyc10g084370.1.1 | MYB transcription factor | 9.97 | 0.69 | -3.84 |  | 24.30 | 3.71 | -2.70 |
| Solyc11g066100.1.1 | heat shock protein | 5.08 | 1.04 | -2.27 |  | 21.93 | 1.03 | -4.40 |
| Solyc12g013620.1.1 | NAC domain protein IPR003441 | 27.85 | 2.94 | -3.24 |  | 63.08 | 1.94 | -5.02 |

**^a^** the value of RPKM.

**^b^** the value of Log_2_(fold-change).
