# Supplementary material for: Comparative Transcriptome Analysis of Resistant and Susceptible Tomato Lines in Response to Infection by Xanthomonas perforans Race T3
Source: Front Plant Sci. 2015 Dec 24;6:1173. doi: 10.3389/fpls.2015.01173 (PMC4689867; doi:10.3389/fpls.2015.01173)
Supplement: Table S4 — Gene ontology (GO) analysis of up-regulated genes for biological process in tomato lines PI 114490 and OH 88119 at 6 d post-inoculation. [file Table4.DOCX]

**Table S4** Gene ontology (GO) analysis of up-regulated genes for biological process in tomato lines PI 114490 and OH 88119 at 6 d post inoculation.

| **GO term** | | **GO name** | **No. of DEGs** | **FDR corrected *p*-value** |
| --- | --- | --- | --- | --- |
| **OH 88119** | | | | |
| GO:0009611 | Response to wounding | | 13 | 7.24e-10 |
| GO:0009605 | Response to external stimulus | | 13 | 7.24e-10 |
| GO:0055114 | Oxidation reduction | | 34 | 1.65e-03 |
| GO:0006950 | Response to stress | | 20 | 5.53e-03 |
| GO:0009607 | Response to biotic stimulus | | 7 | 3.37e-02 |
|  | | | | |
| **P I114490** | | | | |
| GO:0044036 | Cell wall macromolecule metabolic process | | 11 | 2.79e-03 |
| GO:0009415 | Response to water | | 5 | 4.8e-02 |
