# Supplementary material for: Comparative Transcriptome Analysis of Resistant and Susceptible Tomato Lines in Response to Infection by Xanthomonas perforans Race T3
Source: Front Plant Sci. 2015 Dec 24;6:1173. doi: 10.3389/fpls.2015.01173 (PMC4689867; doi:10.3389/fpls.2015.01173)
Supplement: Table S5 — Sets of common differentially expressed genes in RNA-seq analysis with previous cDNA-AFLP results. [file Table5.DOCX]

**Table S5** Sets of common differentially expressed genes in RNA-seq analysis with previous cDNA-AFLP results.

|  |  | **Log_2_ Fold change^b^** | |
| --- | --- | --- | --- |
| **Gene** | **Annotation** | **PT6d vs PM** | **OT6d vs OM** |
| Solyc10g055800.1.1 | Chitinase**^a^** | 3.56 | 4.27 |
| Solyc00g071180.2.1 | Cysteine proteinase inhibitor | 2.12 | 4.16 |
| Solyc03g006700.2.1 | Peroxidase | 3.71 | 2.46 |
| Solyc01g105070.2.1 | peroxidase^a^ | 4.36 | 2.58 |
| Solyc04g079730.1.1 | Allene oxide synthase **^a^** | 2.05 | 2.17 |
| Solyc03g007240.2.1 | Spermidine synthase 1**^a^** | 1.00 | 2.09 |
| [Solyc04g015970.2.1](http://solgenomics.net/feature/view/id/17790507) | ATP-binding cassette 1**^a^** | 2.79 | 1.00 |
| Solyc05g050120.2.1 | Malic enzyme | 2.00 | 1.06 |
| Solyc08g068710.1.1 | N-acetyltransferase | 3.14 | 1.54 |
| Solyc08g074680.2.1 | Polyphenol oxidase | 4.04 | 4.76 |
| Solyc03g122190.2.1 | Jasmonate ZIM domain 2**^a^** | 1.97 | 1.82 |
| Solyc11g068940.1.1 | U-box domain-containing protein 24**^a^** | 7.14 | 5.75 |
| Solyc04g010250.2.1 | Lipase-like protein | 2.11 | 1.51 |
| Solyc07g049530.2.1 | 1-aminocyclopropane-1-carboxylate oxidase**^a^** | 5.19 | 5.47 |

**^a^** differentially expressed gene validated by RT- or qRT-PCR in previous experiment [18].

**^b^**PM: PI 114490 mock-treatment with the sterile solution containing 10 mM MgSO_4_.7H_2_O and 0.025%(v/v) Silwet L77. PT6h: 6 h after inoculation with bacterial spot race T3 in PI 114490. PT6d: 6 day after inoculation with bacterial spot race T3 in PI 114490. OM: OH 88119 mock-treatment with the sterile solution containing 10 mM MgSO_4_.7H_2_O and 0.025%(v/v) Silwet L77. OH6h: 6 h after inoculation with bacterial spot race T3 in OH 88119. OH6d: 6 day after inoculation with bacterial spot race T3 in OH 88119.
