# Supplementary figures and images for: Comparative Transcriptome Analysis of Resistant and Susceptible Tomato Lines in Response to Infection by Xanthomonas perforans Race T3
Source: Front Plant Sci. 2015 Dec 24;6:1173. doi: 10.3389/fpls.2015.01173 (PMC4689867; doi:10.3389/fpls.2015.01173)

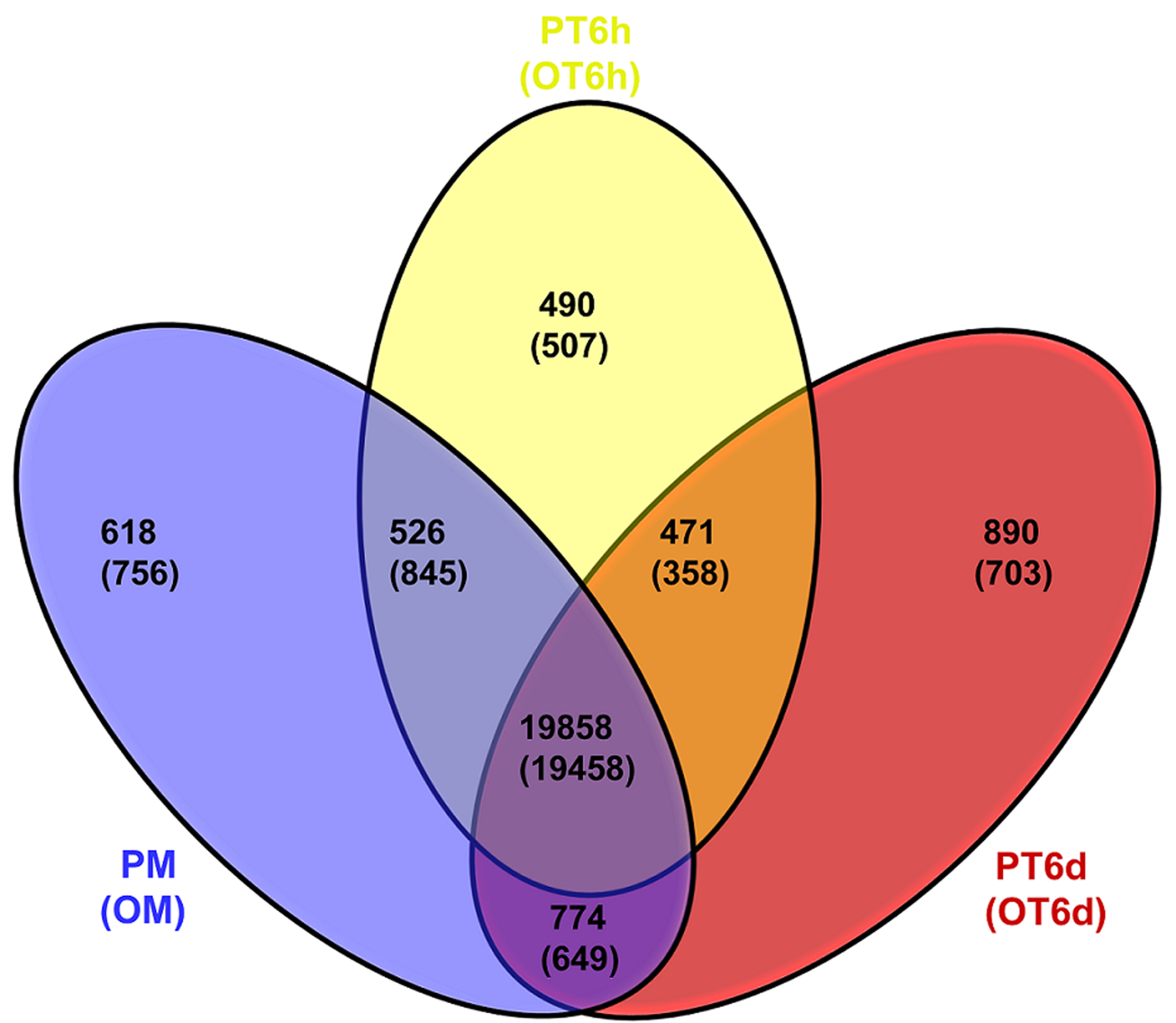

Supplement: Image S1 — Venn diagram showing the overlaps and specific expressed genes between mock samples and samples spray-inoculated with Xanthomonas perforans race T3 in tomato lines PI 114490 and OH 88119. PM and OM: PI 114490 and OH 88119 respective mock-treatment with the sterile solution containing 10 mM MgSO4.7H2O and 0.025%(v/v) Silwet L77. PT and OT: inoculation with bacterial spot race T3 in PI 114490 and OH 88119, respectively. [file Image1.TIF]

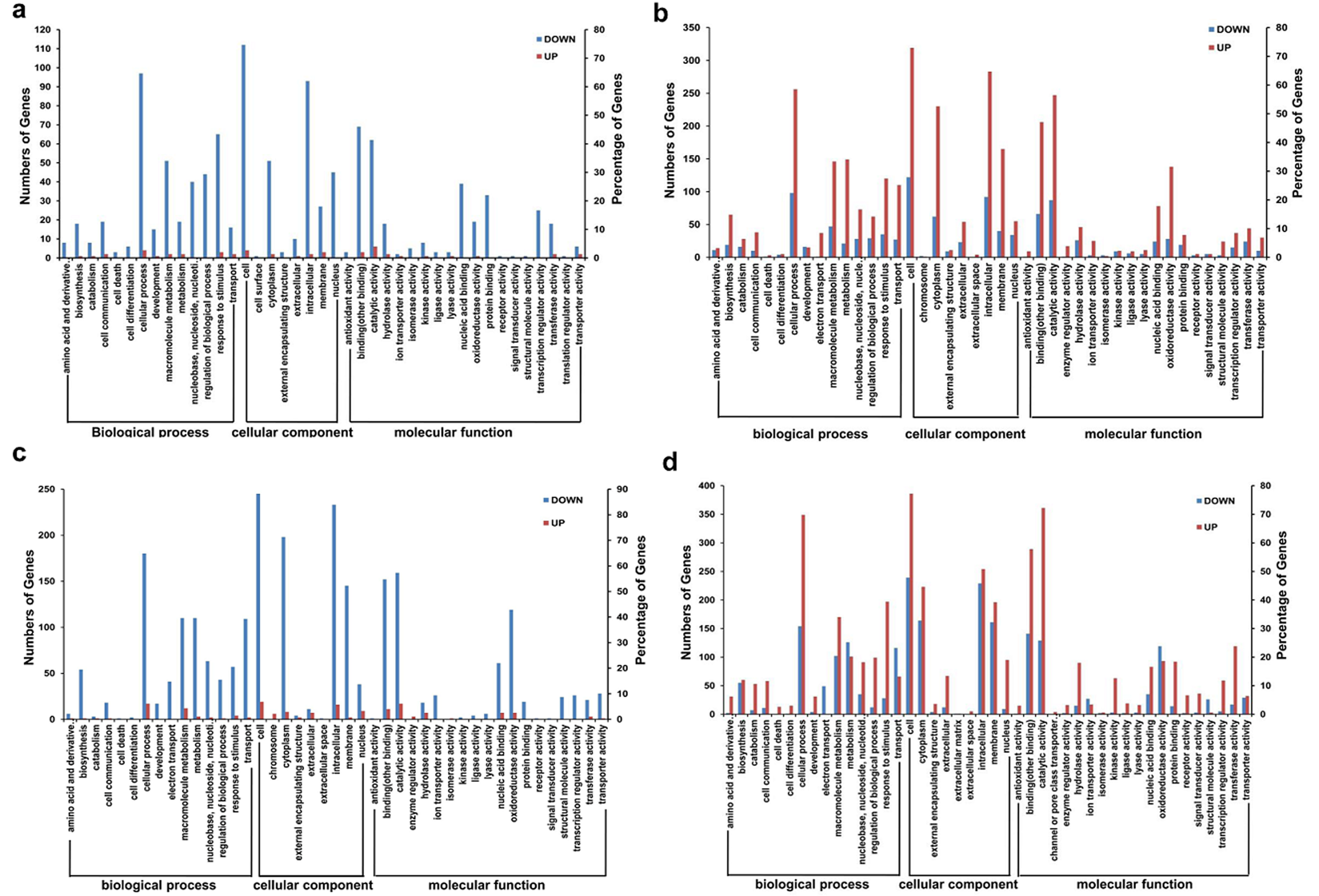

Supplement: Image S2 — Distribution of the differentially expressed genes (DEGs) within Go secondary categories of molecular function, cellular component and biological process. (A) Go classification for DEGs identified from OT6h vs. OM. (B) Go classification for DEGs identified from OT6d vs. OM. (C) Go classification for DEGs identified from PT6h vs. PM. (D) Go classification for DEGs identified from PT6d vs. PM. PM and OM: PI 114490 and OH 88119 respective mock-treatment with the sterile solution containing 10 mM MgSO4.7H2O and 0.025%(v/v) Silwet L77. PT and OT: inoculation with bacterial spot race T3 in PI 114490 and OH 88119, respectively. [file Image2.TIF]
